# Software solutions for evaluation and visualization of laser ablation inductively coupled plasma mass spectrometry imaging (LA-ICP-MSI) data: a short overview

**DOI:** 10.1186/s13321-019-0338-7

**Published:** 2019-02-18

**Authors:** Ralf Weiskirchen, Sabine Weiskirchen, Philipp Kim, Robert Winkler

**Affiliations:** 1 Institute of Molecular Pathobiochemistry, Experimental Gene Therapy and Clinical Chemistry (IFMPEGKC), RWTH University Hospital Aachen, 52074 Aachen, Germany; 2 Department of Biochemistry and Biotechnology, Center for Research and Advanced Studies (CINVESTAV) Irapuato, Km. 9.6 Libramiento Norte Carr. Irapuato-León, 36824 Irapuato, Gto. Mexico; 3 Mass Spectrometry Group, Max Planck Institute for Chemical Ecology, Hans-Knöll-Straße 8, 07745 Jena, Germany

**Keywords:** Visual basic, Application, Excel, Big data, Software, LA-ICP-MS(I), Mass spectrometry imaging, Data formats, Bio-imaging, Data visualization

## Abstract

Mass spectrometry imaging (MSI) using laser ablation (LA) inductively coupled plasma (ICP) is an innovative and exciting methodology to perform highly sensitive elemental analyses. LA-ICP-MSI of metals, trace elements or isotopes in tissues has been applied to a range of biological samples. Several LA-ICP-MSI studies have shown that metals have a highly compartmentalized distribution in some organs, which might be altered in consequence of genetic diseases, intoxication, or malnutrition. Although metal imaging by LA-ICP-MSI is an established methodology, potential pitfalls in the determination of metal concentrations might result from erroneous calibration, standardization, and normalization. In addition, for simple display of final imaging results, most LA-ICP-MSI users prefer to process their measurements by commercial processing software. Such programs typically visualize the regional metal differences in colorful and vivid imaging maps, but might not represent the actual signal densities correctly. There is a great abundance of such MSI data processing programs available differing in quality, usability, integrated features, workflow, reliability, system requirements, speed of data processing, and price. Some software packages contain a multitude of features which are superfluous for most users. In contrast, often only few data formats are used, in case of commercial programs even only the instrument provider’s own raw data format. Therefore, first time and average users are often confused and helpless in choosing the correct software for processing their data. Here we have briefly summarized software packages, data routines, macros, programming tools, scripts, algorithms, or self-written patches and updates for existing programs presently in use for mining LA-ICP-MSI data.
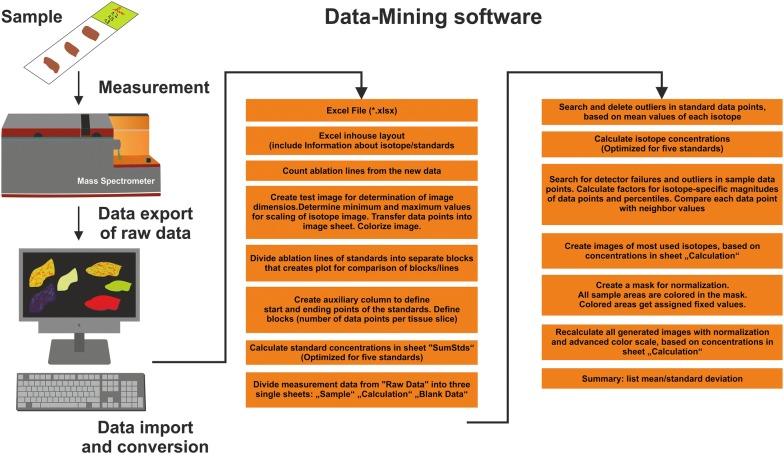

## Introduction

Inductively coupled plasma (ICP) mass spectrometry (MS) has become a routine method for the accurate determination of the elemental composition of complex samples [1]. In combination with laser ablation (LA), surfaces can be sampled with high lateral resolution, to generate ‘elemental maps’. LA-ICP-MS imaging (LA-ICP-MSI) of organic tissues was adopted for biological and medical research and provides new insights into physiological processes during disease [2].

However, for producing the final images, vast amounts of raw data need to be processed. The sample is scanned by the laser line-by-line. The material is continuously ablated during the movement and analyzed by the ICP-MS system. Mass spectra of the different sampling localizations are either written as separate data files or as a continuous data stream into a single file.

**Fig. 1:**
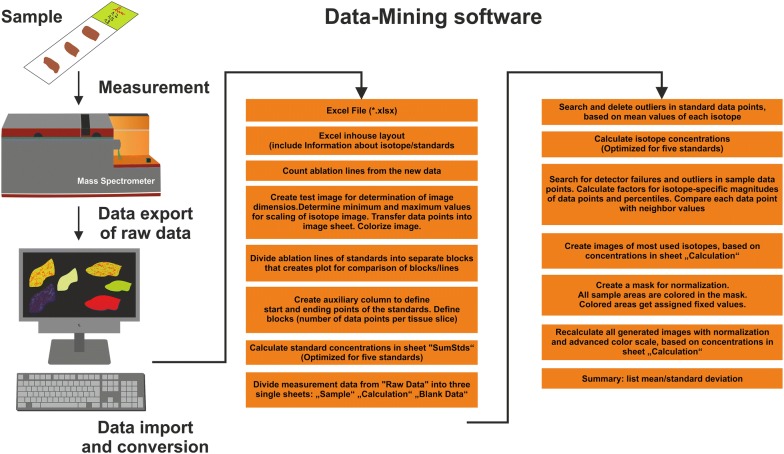
Principal workflow of creating element maps from mass spectrometry imaging data. During MS analysis, more or less structured raw data is generated from the measurement of a sample. In a first step, this data is converted into a format that is readable for the chosen analysis software. In ELAI for example this format is an Excel File with an in-house format. Subsequently, the list of data is splitted into individual rows corresponding to the measurements of the individual line-by-line scans. Thereafter, the measured values of each element are normalized and transferred into absolute concentrations. In a final step, the calculated concentrations are reconstructed into a final element map. Some of the necessary working steps during data-mining from Excel file to image and mean concentrations and are listed

In both cases, this requires a wealth of processing steps in which the informative signals have to be extracted, assembled to a location-signal data matrix and visualized as a final colored 2D-image reflecting the spatial distribution and concentration of the measured elements (Fig. 1).

**Fig. 2:**
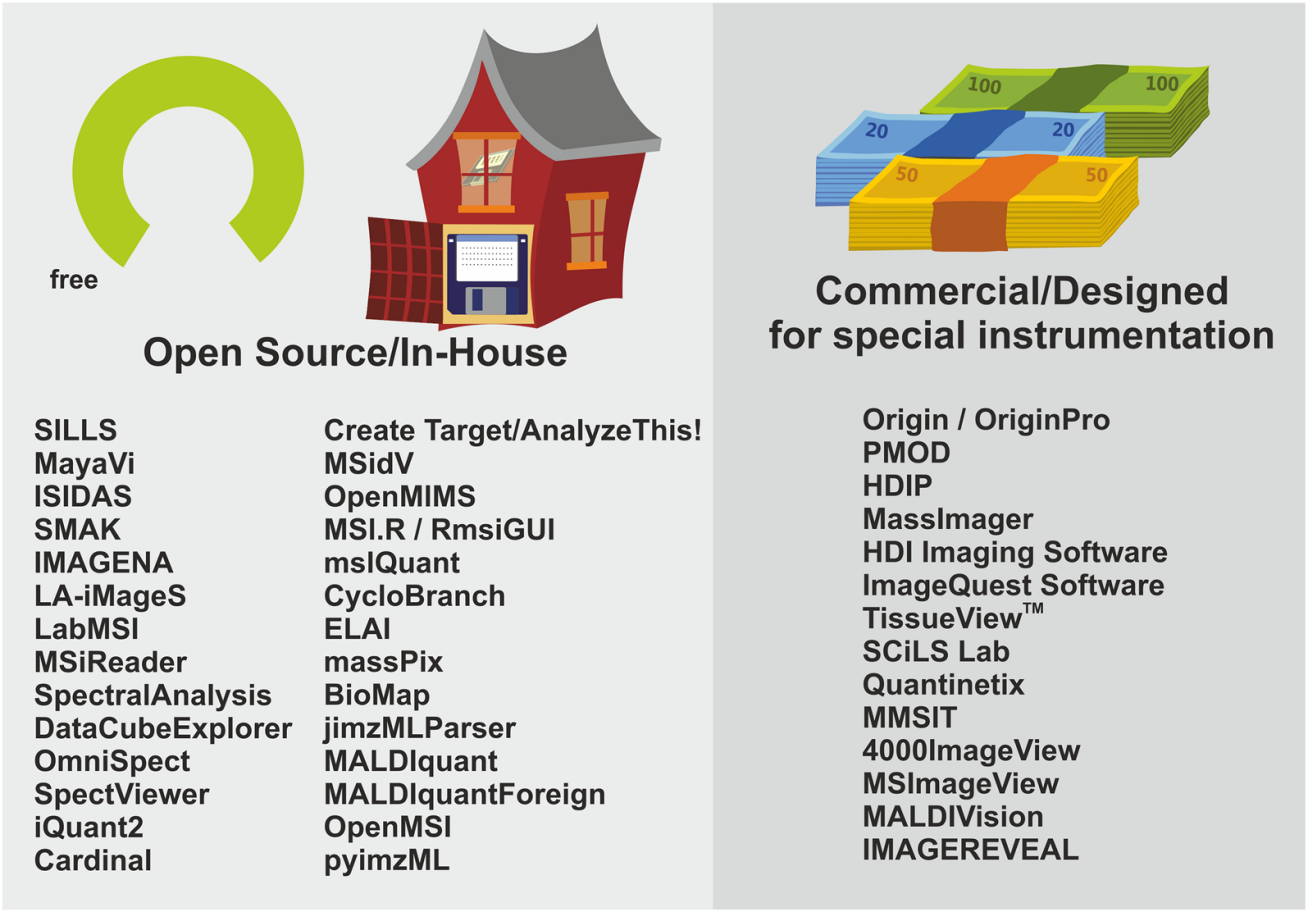
Software used in the field of LA-ICP-MS imaging. There is a number of open source and in-house software available. On the other hand, several copyright-protected commercial available software solutions are frequently used in analyzing LA-ICP-MS data sets

The raw LA-ICP-MSI data are either device-dependent, sometimes even binary encoded, or stored in a more common format such as simple text tables. In most cases, these lists have to be edited and reformatted to allow the export to external applications for further analysis, or the generation of high-quality elemental distribution bio-images. This can be very labor-intensive. The time for translating raw data to a meaningful image can by far exceed the time for data acquisition [3]. Hence, there has been an increasing interest in the generation of tools for post-processing LA-ICP-MSI data and for automated generation of such images during the last years. Consequently, a large abundance of such programs was developed (Fig. 2). However, the wealth of free open source applications, in-house software developments, or commercially distributed programs customized or partly adapted to special devices cause confusion among first-time users.

Actually, there is a plethora of software available used for LA-ICP-MSI data mining. To identify software solutions presently used for evaluation of LA-ICP-MS data, we have screened the MEDLINE database for abstracts using the search terms “LA-ICP-MS” or “mass spectrometry imaging”. The search term “LA-ICP-MS” was found in 509 articles; while the search term “mass spectrometry imaging” resulted in the identification of 1275 articles (a new search was carried out at the time of the revision of this article on December 26, 2018). In our review, we will discuss some of the most widespread programs, which are frequently used in publications. In addition, we will highlight some novel innovative software routines written by well-experienced users for processing of special LA-ICP-MS applications.

## MSI and LA-ICP-MSI data formats

The first requirement of any data processing workflow is the readability of the input data. In mass spectrometry imaging (MSI), two types of information are necessary: (1) The sampling point, i.e. the localization of each pixel and (2) the spectral information of each sampling point. For 2D images, the localization data can be a simple text table with values representing the x and y position of each point. However, for mass spectrometry data a large variety of different file formats are used. Each provider of MS devices encodes the raw data in a different binary encoded format, which is not readable without suitable libraries. Thus, several providers of LA-ICP-MSI and other MSI techniques provide software which is designed for their devices. Naturally, the possibilities to exchange and re-process data in proprietary formats it limited. Some vendor format libraries have been released to the public [4, 5]. But in general, their distribution and use is restricted, which impedes their integration in community software projects.

To promote the sharing of raw mass spectrometry data, the HUPO Proteomics Standards Initiative (PSI) [6] defined the mzML file format [7]. Most MS data processing programs are able to read and write in mzML format. Therefore, this format should be used whenever possible. MS data from common MS instruments can be converted to mzML using the ProteoWizard [5]. With the ProteoWizard also other formats such as Mascot generic files or table/text can be generated, which makes it an indispensable tool in MS data processing. The processing of MS data with the popular high-level programming language Java [8], Python [9] and R [10], has been made possible by different libraries (Table 1). In the case of LA-ICP-MS, the export of tabular data is common.

**Table 1:** Available software for the processing and visualization of laser ablation inductively coupled plasma mass spectrometry imaging (LA-ICP-MSI)

Program	Main function	Data formats	Platform	Programming language	Version/release or publication date^a^	License	Open source	Free of cost	Project webpage^b^/ References
4000 Series Imaging	MSI data acquisition, DFI	4000 Series instrumentation from Applied BMac OSystems	Windows	.NET	V 3	Novartis and Applied Biosystems	No	REG	https://ms-imaging.org/wp/4000-series-imaging/ [73]
BioMap	Visualization of biological imaging data, multiple data types (MSI, optical, CT etc.) are supported	Analyze 7.5, imzML	Mac OS, Linux, Windows	IDL™	V 3.8.0.4, 2016-09-11	Novartis	No	REG	https://ms-imaging.org/wp/biomap/ [22]
CreateTarget/AnalyzeThis!	MSI data acquisition, DFI	Bruker	Windows	Visual Basic 6	2006-09-12	University of Leuven (Belgium)	No	Yes	https://ms-imaging.org/wp/createtargetanalyzethis/ [64, 91]
Cardinal	Preprocessing, statistics, spatial segmentation and visualization of MSI data	Analyze 7.5, imzML	Mac OS, Linux, Windows	R	2018-12-18	Artistic-2.0	Yes	Yes	http://cardinalmsi.org/ [67, 92]
CycloBranch	Analysis of MSI data with special focus on dereplication of organic compounds	Bruker, Thermo, Waters, mzML, mzXML, imzML, mgf, txt	Mac OS, Linux, Windows	C++	2018-11-18	GPL	Yes	Yes	https://ms.biomed.cas.cz/cyclobranch/docs/html/index.html [71, 72, 93]
DataCube Explorer	Visualization of large MSI data sets	Analyze 7.5, imzML, AMOLF	Windows	NET 4	V 2.3.0.0, 2014-05-27	AMOLF	No	REG	https://amolf.nl/download/datacubeexplorer [62, 63]
ELAI—Excel-based Laser-Ablation Imaging	Generation, analysis and visualization of LA-ICP-MSI data	Tabular	Windows	Microsoft Excel with Visual Basic for Applications (VBA)	2016-02-05	NLD	Yes	AUT	AUT [23]
HDIP	Processing and visualization of LA-ICP-MSI data	HDF5, raw instrument data (not further specified)	Windows	Proprietary	2018-07-03	Teledyne	No	No	http://www.teledynecetac.com/products/laser-ablation/hdip-imaging-software [74]
HDI—High Definition Imaging	Processing and visualization of multimodal Waters MSI data and statistics, DFI	Waters	Windows	Proprietary	V 1.4, 2016-06	Waters	No	No	http://www.waters.com/waters/en_US/High-Definition-Imaging-%28HDI%29-Software/nav.htm?cid=134833914&&locale=us_EN [94]
IMAGEREVEAL	Statistical analyses, quantification, comparison with reference images, refinement	Analyze 7.5, imzML, kbd, IMDX	Windows	Proprietary	2018-08-22	Shimzadzu	No	No	https://www.shimadzu.com/an/lifescience/imaging/reveal.html [77]
IMAGENA—Image Generation and Analysis	Creation and analysis of images from LA-ICP-MS data	Tabular	Mac OS, Linux, Windows	C++,Qt	2011-10-01	NLD	No	AUT	AUT [33]
iQuant2	Creation and analysis of images from LA-ICP-MS data	Tabular	Windows	Visual Basic 2008 Express Edition	2018-02-23	NLD	No	AUT	AUT [35]
ImageQuest	Processing, manipulation and analysis of MSI data, DFI	Thermo	Windows	Proprietary	V 1.0.1, 2009-05	Thermo Fisher Scientific	No	No	https://www.thermofisher.com/order/catalog/product/10137985 [76]
imzML Converter	Creation of imzML files from raw data	mzML, tabular	Mac OS, Linux, Windows	Java	V 1.3.0, 2012-08-30	NLD	No	REG	http://www.cs.bham.ac.uk/~ibs/imzMLConverter/ [95]
ISIDAS—Interactive Spectral Imaging Data Analysis Software	Creation of MSI files	Tabular	Mac OS, Linux, Windows	Python	2010-10-29	NLD	No	AUT	AUT [37]
jimzMLParser	Parsing imzML and mzML files in Java	imzML, mzML	Mac OS, Linux, Windows	Java library	2018-12-18	NLD	Yes	Yes	https://github.com/AlanRace/jimzMLParser [95, 96]
LA-iMageS	Visualization of LA-ICP-MSI data	ELAN XL	Mac OS, Linux, Windows	Java	2017-01-01	GPL	Yes	Yes	http://www.la-images.net/ [3, 40]
LabMSI	Analysis and visualization of MSI data	imzML	Mac OS, Linux, Windows	LabView	2015-07-01	NLD	No	AUT	AUT [41]
MALDIquant, MALDIquantForeign	R library for import and processing of MS(I) data	Analyze 7.5, imzML, tabular	Mac OS, Linux, Windows	R package	2018-11-26	GPL	Yes	Yes	https://github.com/sgibb/MALDIquant/ https://github.com/sgibb/MALDIquantForeign/ [12, 13]
MALDIVision	Processing, visualization and analysis of MSI data	Analyze 7.5, imzML	Mac OS, Windows	Proprietary	V 2.22	Premier Biosoft	No	No	http://premierbiosoft.com/maldi-tissue-imaging/ [14]
MassImager	Data mining/screening in MSI data (advanced statistics) and visualization	ANDI (CDF), ASCII (TXT), Matlab, mzXML	Windows	Proprietary	V 1.0 2018-02-05	Chemmind Technologies	No	No	http://www.chemmind.com/en/support_download.html [66, 67]
massPix	Analysis of MSI data in R, with special focus on lipids	imzML	Mac OS, Linux, Windows	R package	2017-08-08	NLD	Yes	Yes	https://github.com/hallz/massPix [69, 97]
MayaVi	Visualization of scientific data with Python	Python compatible formats	Mac OS, Linux, Windows	Python	2005-09-13	BSD	Yes	Yes	http://mayavi.sourceforge.net/install.html [46, 98]
MMSIT	Control software for Voyager STR and sSTR instruments, DFI	Voyager	Windows	Proprietary	2007-12-02	Novartis	No	REG	https://ms-imaging.org/wp/mmsit/ [82]
MSI.R/RmsiGUI	Visualization and plotting of MSI data, statistics and processing of large data sets on computer clusters	imzML	Mac OS, Linux, Windows	Java, R	2018-12-19	GPL	Yes	Yes	https://bitbucket.org/lababi/msi.r https://bitbucket.org/lababi/rmsigui [68, 99, 100]
MSIdV	Visualization of MSI data	imzML	Windows	Python	V 1.1 2016-04-13	NLD	Yes	Yes	https://sourceforge.net/projects/msidv/ [70, 101]
msIQuant	Quantification and visualization of large MSI data sets	imzML	Windows	C++, .NET	V 2.0.2.14 2016-05-25	Uppsala University	No	REG	https://ms-imaging.org/wp/paquan/ [66, 102]
MSiReader	Analysis of high-resolution MSI data	Analyze 7.5, imzML, mzXML, Tabular	Mac OS, Linux, Windows	MATLAB	V 1.0 2018-01	BSD	Yes	Yes	http://www4.ncsu.edu/~dcmuddim/downloads.html (not working on Jan 8 2019) [47, 49, 50]
OpenMSI	Web platform for management and visualization of MSI data	Analyze 7.5, imzML, raw (various)	Web platform with API	Proprietary	2013-10-02	NERSC	No	REG	https://openmsi.nersc.gov/openmsi/client/ [103, 104]
OmniSpect	Visualization and analysis of MSI data	Analyze 7.5, imzML, mzXML, netCDF	Mac OS, Linux, Windows	MATLAB	2013-02-26	NLD	Yes	Yes	https://cs.appstate.edu/~rmp/omnispect.zip [60, 105]
OpenMIMS	ImageJ plugin for analyzing NanoSIMS 50 and 50L data, DFI	Cameca SIMS	Mac OS, Linux, Windows	Java	2018-04-05	NLD	Yes	Yes	https://github.com/BWHCNI/OpenMIMS [65, 106]
Origin and OriginPro	Scientific data analysis and graphing	Connectors to C, Python, R, MATLAB, LabView, EXCEL	Windows	Proprietary	2018-01-10	OriginLab	No	No	https://www.originlab.com/index.aspx?go=Products/Origin [84, 107]
PMOD	Biomedical imaging and quantification, DFI	Clinical and (pre)clinical systems	Mac OS, Linux, Windows	Java	NA	PMOD Technologies LLC	No	No	https://www.pmod.com/web/ [86]
pyimzML	Library for parsing MSI data with Python	imzML	Mac OS, Linux, Windows	Python library	2018-09-20	Apache-2.0	Yes	Yes	https://github.com/alexandrovteam/pyimzML [108]
Quantinetix	Quantification and visualization of MSI data	Bruker, Sciex, Thermo, Waters, imzML	Mac OS, Linux, Windows	Proprietary	NA	ImaBiotech	No	No	https://www.imabiotech.com/quantinetix-mass-spec-imabiotech/ [109]
SCiLS Lab MVS	Visualization and analysis of MSI data	Bruker, imzML	Windows	Proprietary	V 7.01.10764	SCiLS Lab	No	No	https://scils.de/download/ [90]
SILLS—Signal Integration for Laboratory Laser Systems	Data reduction and quantification of LA-ICP-MSI data	ELAN XL	Windows	MATLAB, EXCEL	NA	ETH Zürich	No	REG	http://www.orefluids.ethz.ch/software/sills.html [54]
SMAK—Sam’s Microprobe Analysis Kit	Analysis toolkit for imaging data; initially for x-ray microprobes	Tabular	Mac OS (wine), Windows	Python	V 1.50 2018-11-10	SMAK	Yes	Yes	https://www.sams-xrays.com/smak [57]
SpectralAnalysis	Analysis and visualization of MSI data, statistics	imzML	Mac OS, Linux, Windows	C, Java, MATLAB	2018-10-30	Apache-2.0	Yes	Yes	https://github.com/AlanRace/SpectralAnalysis [58, 110]
SpectViewer	Visualization of MSI data, suitable for very large data files without binning	imzML	Linux, Windows	Proprietary	2014-10-07	NLD	No	AUT	https://ms-imaging.org/wp/imzml/software-tools/cea-spect-viewer/ [61, 111]
TissueView	Visualization and manipulation of MSI data, export of images	Applied BMac OSystems	Windows	Proprietary	V 1.1, 2010-05-21	ThermoFisher/Applied Biosystems/MDS Sciex, DFI	No	No	https://assets.thermofisher.com/TFS-Assets/LSG/Warranties/cms_052419.pdf [112]

Apache-2.0—Apache License 2.0 (https://www.apache.org/licenses/), Artistic-2.0—Artistic License 2.0 (https://opensource.org/licenses/Artistic-2.0), AUT—Authors that have established this software need to be contacted to obtain respective software and further information, BSD—BSD License (https://opensource.org/licenses/bsd-license.html), DFI—designed for special instrumentation, GPL—GNU General Public License (https://www.gnu.org/licenses/gpl.html), MSI—Mass Spectrometry Imaging, NA—No information accessible, NERSC—National Energy Research Scientific Computing Center (USA), NLD—No license defined, REG—requires registration, license agreement or personal data, SMAK—SMAK License (https://www.sams-xrays.com/license)

^a^In case of missing versions/release dates, the date of a relevant publication is listed

^b^Webpages were last accessed February 6, 2019

For MSI, the most accepted community format has become imzML [11]. ImzML files can be created from raw mzML files with the free software tool imzML Converter [12] or from tabular data, e.g. using R with the MALDIquant/MALDIquantForeign packages [13, 14]. However, also the Analyze 7.5 (Mayo Clinic in Rochester, MN, USA) as well as simple text formats are commonly used. In general, LA-ICP-MSI data sets can be processed, analyzed and visualized with already available tools and programs.

As a minimal requirement, any MSI program should be able to read at least one open community format, since this is the fundamental for data exchange and reproducibility of data workflows on different platforms. Imaging data sets can be achieved in public repositories such as PRIDE [15] and Zenodo [16]. Obviously, the re-use of MSI data is promoted by using community data formats.

A short overview of the discussed software including information about supported file formats, platforms, terms of license, references and availability is given in Table 1.

## Examples of free and open-source software used in LA-ICP-MS imaging

There is a large number of free and open-source software, as well as in-house software that is presently used for LA-ICP-MS data mining.

Ideally, the code of published programs should be made public (https://codeisscience.github.io/manifesto/manifesto.html) and released under the terms of a license that promotes further community-driven development, such as the General Public License GPL [17]. Public repositories such as Bitbucket [18], GitHub [19] or SourceForge [20] provide excellent platforms for the collaborative software development. Nevertheless, for several freely available programs listed in Table 1 the source code is not publicly accessible, or their license status is unclear. Sometimes a registration is necessary, or the authors of the software have to be contacted, which complicates the testing of these programs.

Some of the non-commercial programs provide a similar user experience as commercial software: They are elaborately designed, include sophisticated routines for quantitative data processing, offer features for handling of calibration factors, or contain extensive statistic interfaces. The features of some of these open programs, data routines, macros, and programming tools will be briefly discussed in the following.

## BioMap

BioMap (Novartis, Basel, Switzerland) was originally developed for the evaluation of MRI data and was subsequently adapted to many other imaging applications including position emission tomography, computed tomography, near-infrared fluorescence imaging, and mass spectrometry imaging (MSI). It is written in Interactive Data Language (IDL) that is preferentially tailored to the needs of scientists, engineers and developers. Additionally, BioMap contains a large variety of functions for visualizations of imaging data. These are particularly suited to process single-subject-data, combine results from several subjects or sessions, and to document the final result of a study. Therefore, BioMap is comprehensive standalone software, which does not require other software-tools. It runs on many different systems (Windows, Unix, Linux, MAC OS) and therefore offers many application possibilities [21]. A free download, including a comprehensive description of the program, information about its setup, tutorials, and demo data sets is available at [22].

## ELAI

**Fig. 3:**
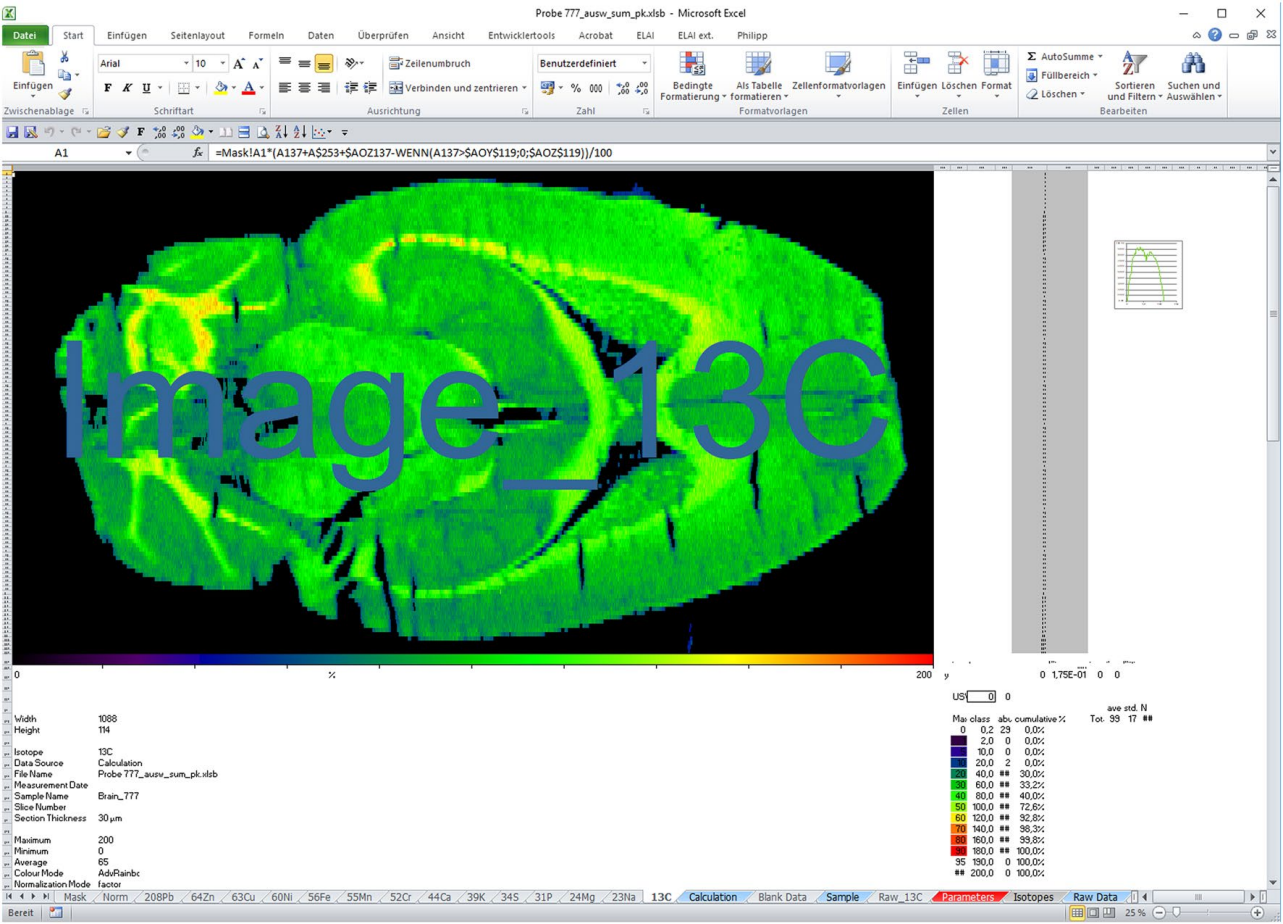
Screenshot of Excel Laser Ablation Imaging (ELAI). ELAI is a modularly constructed software tool running under Windows designed for reconstructing element distribution maps using Microsoft Excel with the aid of Visual Basic for Applications (VBA) user-defined functions. In the depicted example, a murine brain section was analysed for content of ^13^C. Details about this software tool were published elsewhere [23]

Excel Laser Ablation Imaging (ELAI) was first introduced by our lab in 2016 [23]. It was designed as a modularly constructed software tool for reconstructing element distribution maps using Microsoft Excel with the aid of Visual Basic for Applications (VBA) user-defined functions. The integration into the Microsoft Windows (Fig. 3) enables easy adaptation to special requirements and simple transfer to other systems. Its simplicity further facilitates the quick generation of high-resolution images that can be exported to several common image formats (JPEG, TIFF), which can be depicted in different pseudo-color scales [24–32]. ELAI has an overall simple workflow in data analysis and contains special functions and features for calibration, de-spiking, and image export. Importantly, it is possible to read-out absolute concentrations from regions of interest. Disadvantages of ELAI are the need of a Windows license for operating and the slightly decelerated processing time, when analysing large data sets that is caused by the processing of the VBA macros. The open-source standalone application with complete source code and comprehensive documentation is freely available and downloadable from the supplementary material of [23]. Based on its modular construction, ELAI can be easily customized for many other applications by adding new or modifying existing macros. However, also this software has some disadvantages, which particularly concerns speed when analyzing large data sets. Therefore, ELAI is under constant improvement in our laboratory and new releases of ELAI are available on request.

## IMAGENA

IMAgeGENeration and Analysis (IMAGENA) is an interactive C++ based software tool, specially developed for speeding up post-processing of LA-ICP-MSI data [33]. Therefore, it is optimised for LA-ICP-MSI applications and suitable to easy handle raw data generated by this technology. IMAGENA allows generation of images from a continuous list of raw data points and conversion of these into other commonly readable image file formats. It protrudes through its simplicity and easy-to-use graphical user interface. Most importantly, it includes tools for calibration and correction of signal drifts in the y-direction. Data can be visualized in either greyscale or pseudo-colours [34]. Moreover, with IMAGENA it is possible to scale, smooth, and read-out of average signals within free-hand drawn regions of interest, enhance contrast of images, and allows data interpolation as well as precise calibration [33].

## iQuant2

**Fig. 4:**
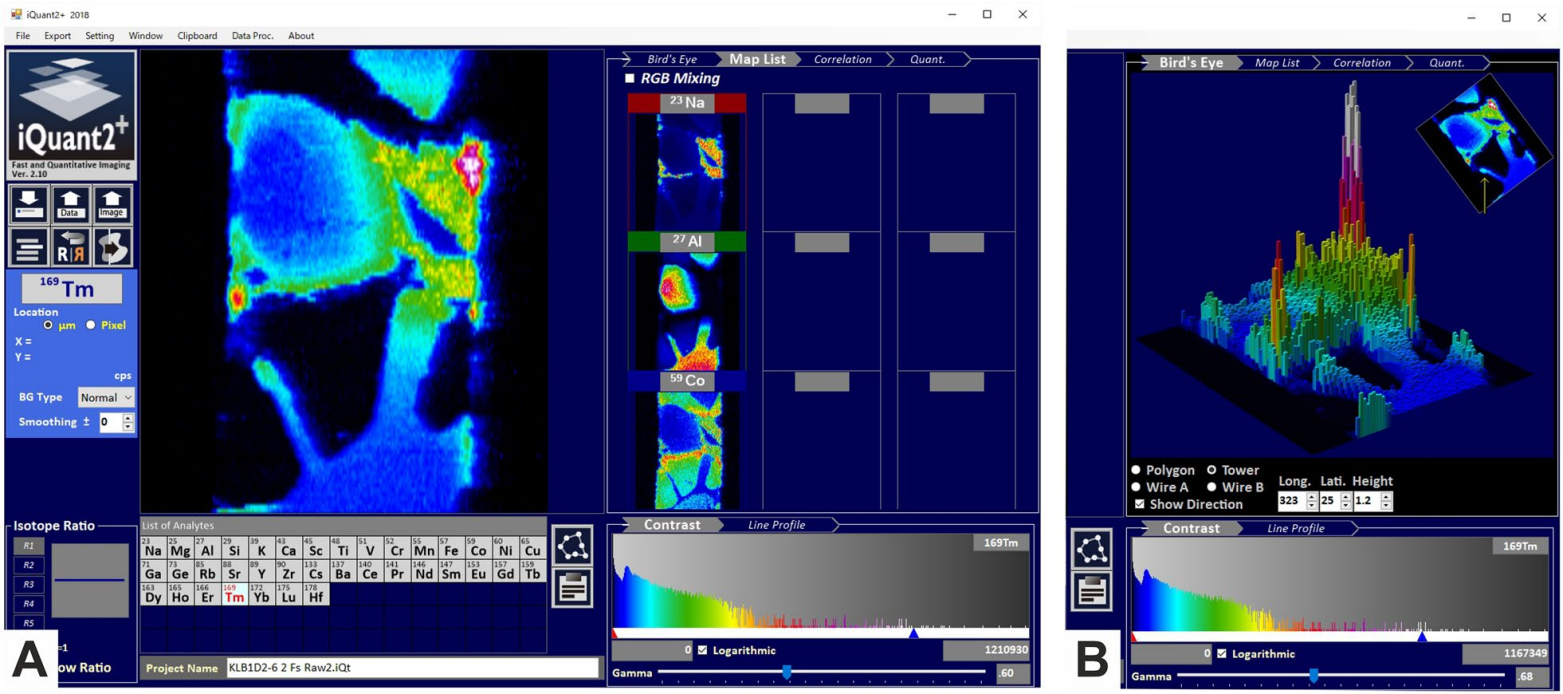
iQuant2 for visualization images from signal intensity data obtained by LA-ICP-MS. **A** The main window of iQuant2 contains well-structured panels, including an image panel, image list panel, ratio panel, list of selected isotopes, and a contrast adjustment option. **B** iQuant further contains an “Bird’s Eye View Panel” option, in which the constructed objects can be shown either as a polygon or a wireframe in which the intensity of each point is expressed as the height of the tower. This figure was kindly provided by Dr. Takafumi Hirata (Division of Earth and Planetary Sciences, Kyoto University, Kitashirakawa Oiwake-cho, Kyoto, Japan). Details about this software program can be found elsewhere [35]

iQuant2 was recently developed in Windows 7 OS with the Visual Basic 2008 Express Edition [35]. This software runs on Windows 8 and 10 and has a mouse-controlled graphical interface with click options, scrolling wheel, and drag-and-drop options. Quant2 has a clear and pleasing layout (Fig. 4A). The main window of iQuant2 contains well-structured panels, including an image panel, image list panel, ratio panel, list of selected isotopes, and a contrast adjustment option. Noteworthy is the integrated, innovative ‘Bird’s Eye View Panel’, in which the constructed 3D objects can be shown either as a polygon or a wireframe (Fig. 4B). The program allows image shape correction, RGB mixture, and line profile analysis between two data points [36]. In iQuant2 it is further possible to visualize the correlation between signal intensities of isotopes and to perform a semi-quantitative analysis. Like in ELAI, it is possible to export images to a number of different formats.

## ISIDAS

The Interactive Spectral Imaging Data Analysis Software (ISIDAS) is written in the Python programming language. It was originally developed as an in-house software [37]. It allows quick data reduction and is therefore highly suitable to quantify spatial and regional element distribution reconstructed from tissue cut into serial consecutive sections [38]. Since ISIDAS and MayaVi are based on the same programming language, images can be easily exported into the MayaVi and MayaVi2 programs.

## LA-iMageS

**Fig. 5:**
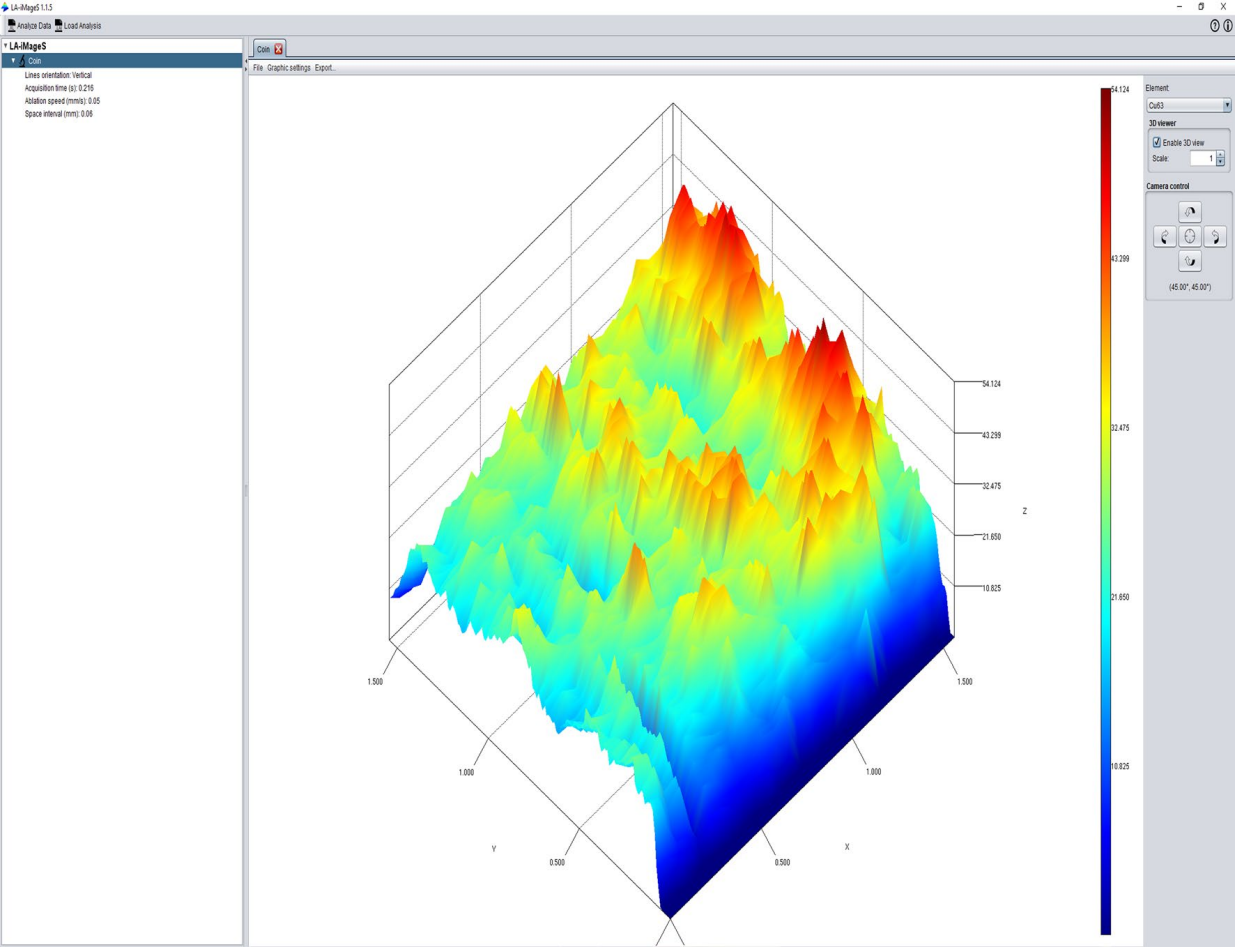
The basic graphical control element in the LA-iMageS software. The program was downloaded and installed following the instructions provided. Thereafter, the example file “Coin/CU63” with activated 3D viewer was loaded and a screenshot taken. Details about this program are given elsewhere [3]

LA-iMageS is an open-source, free-to-use, multiplatform Java standalone application for generation of 2D/3D images from ICP-MS data [3]. It is particularly suitable for fast and automatic generation of high-quality elemental distribution bioimaging maps from LA-ICP-MS data in PerkinElmer XL format. The program has manifold possibilities to customize the elemental distribution images (e.g. colour, resolution, and 2D/3D visualization) and convinces the user by its easy specification of data acquisition parameters (ablation speed time, acquisition time) and data line positions (Fig. 5) [39]. The LA-iMageS application has a clear layout for elemental data extraction, data visualization, and data export. A download for LA-iMageS including a quick-start tutorial is available [40].

## LabMSI

LabMSI is an in-house software developed in the lab of Takahashi et al. [41]. It was created in the system-design LabView platform and development environment released from National Instruments Corporation (Tokyo, Japan). It can be used for targeted and non-targeted imaging MS analyses. LabMSI runs on Windows, Linux, and Mac OS systems and allows handling large imzML files over 100 GB. Moreover, it can calculate average mass spectrums that can be depicted as spatial maps. In addition, the user can define several regions of interest (ROI) to investigate differences in average spectra of ROI. In principle, there is no limitation to the imaging MS data capacity to be analysed involving any profile spectrum and line spectrum data in imzML format [41]. More information about this software and a download link (that was unfortunately not working at the time during setting up this review) are given in the original publication [41].

## MayaVi

MayaVi, meaning magical in Sanskrit, is a free open source software written in the general-purpose, easily readable programming language Python [42]. MayaVi was originally introduced in 2001 [43] and runs on various operating systems, has a pipeline-based architecture and includes a number of useful modules including a graphical user interface allowing easy handling of data. The program supports a large number of visualization algorithms and contains powerful image manipulation algorithms. In MayaVi, multiple data sets can be loaded simultaneously and displayed in variable output formats [44]. Furthermore, the program is highly helpful in visualization of three-dimensional computational fluid dynamics. The usage of the visualization toolkit written in C++ permits to bind this program to programming languages including Tcl, Python, and Java. A standalone version with solid documentation and multiple upgrades are available for free [45, 46].

## MSiReader

**Fig. 6:**
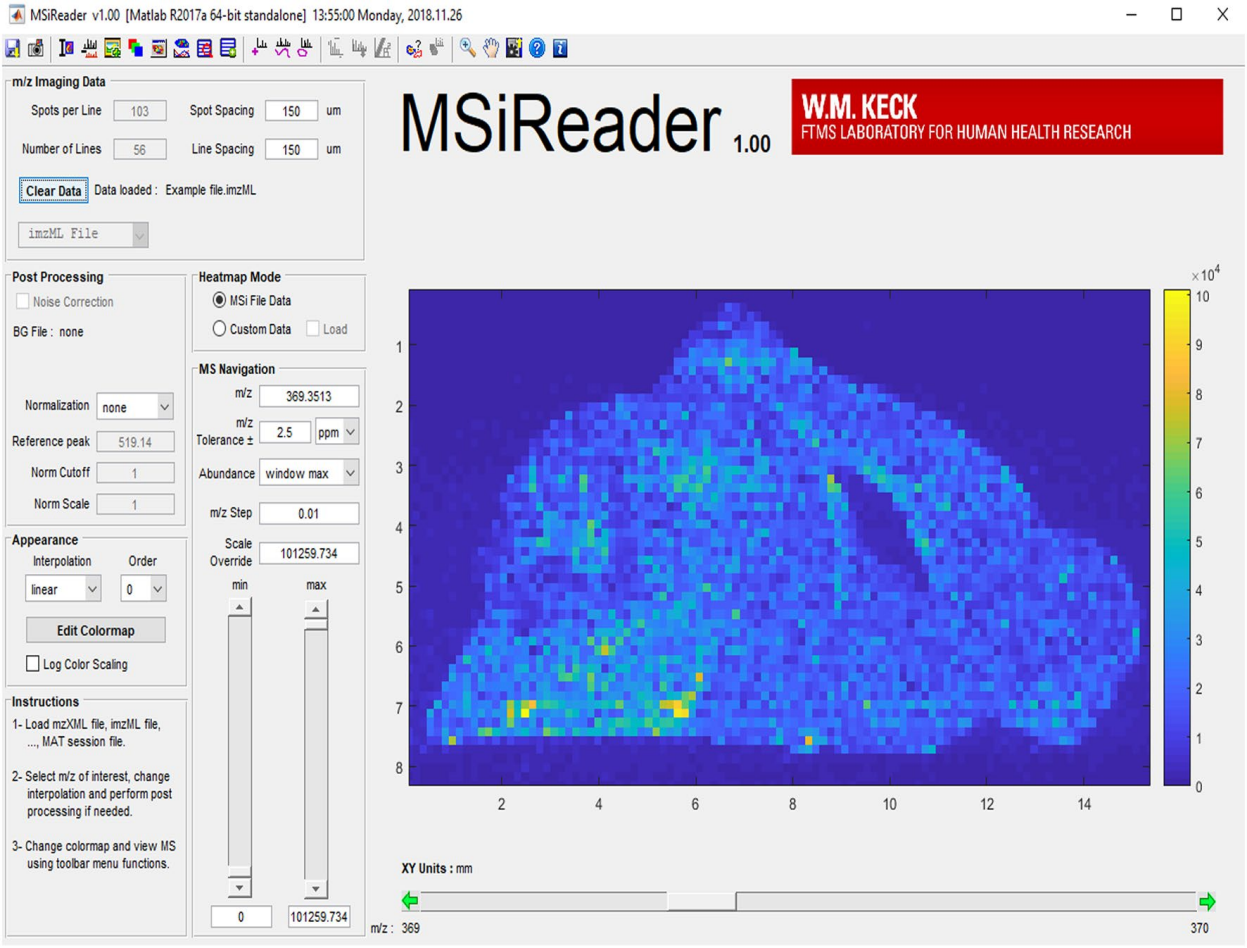
Representative screenshot of the MSiReader software. MSIReader is a MATLAB application with a well-structured organization. Its design is thoughtful which allows easy usage. The program was installed and a not further specified sample file provided by the authors of the software loaded. More information about this software is given elsewhere [47, 49, 50]

MSIReader is an open-source vendor-neutral MATLAB application (Fig. 6), which was released by researchers at the North Carolina State University [47]. A major update of the MSI software offering a multitude of newly added features was recently released [41]. It is capable of analysing most common MSI data formats, can calculate absolute concentrations, and an image overlay feature allows the incorporation of complementary imaging modalities [48]. Most interestingly, MSiReader contains a quality assurance feature to generate mass measurement accuracy (MMA) heatmaps for measured elements. A detailed discussion of the individual MSiReader features with representative colourful screenshots of analysed sample sets is given elsewhere [49]. Moreover, a download link for the program, including full documentation is available at [50].

## SILLS

Signal Integration for Laboratory Laser Systems (SILLS) was software introduced in 2008 and designed for flexible data reduction and concentration calculation of LA-ICP-MS signals [51]. It was developed by MathWorks [52] and written in MATLAB, a multi-paradigm numerical computing environment, allowing matrix manipulation, plotting of functions and data, and implementation of all kinds of algorithms. This permits the program to interface with a variety of other programs, even if they were written in other languages. It is user-friendly because the built-in graphics make it easy to visualize and gain insights from data [53]. SILLS includes options to display raw transient signals, does not require copy-paste steps between spreadsheets, and contains a graphical user interface for signal integration. Moreover, SILLS contains routines for spike/outlier detection, drift correction, and has a variety of quantification possibilities and report writing possibilities. A standalone version, including good documentation is freely available [54].

## SMAK

The Sam’s Microprobe Analysis Kit (SMAK) was originally developed for the processing of X-ray fluorescence microprobe data [55]. It contains different analysis tools for the generation of image maps such as correlation plots, image filtering, multiple image fitting, semi-quantitative element analysis, principle component analysis, dead-time correction, tomographic reconstructions, and math operations on data channels [56]. It convinces the user by a good graphical user interface and keyboard shortcuts. SMAK accepts variable data formats, permits chemical speciation mapping, and is able to rebin data to capture missed peaks. Free downloads of SMAK for Windows and Mac OS, including comprehensive documentation, are available [57].

## SpectralAnalysis

In 2016, SpectralAnalysis was introduced as a program developed with extensibility in mind to stimulate development, comparisons, and evaluation of data analysis algorithms [58]. As such, it has the capability to handle multiple spectral imaging modalities, each of which captures different information about a sample. Moreover, it has a number of features for normalization and also includes tools for principal component analysis, non-negative matrix factorization, autocorrelation, and probabilistic latent semantic analysis [58, 59]. Illustrative examples provided in the original publication demonstrate that this program is able to visualize and process large data sets. The authors further provided details on how to convert data into a suitable format using imzMLConverter and any other information about that program in a Supplementary file published in the mentioned publication.

## Other useful open-source software tools for analyzing and processing of LA-ICP-MS data sets

**Fig. 7:**
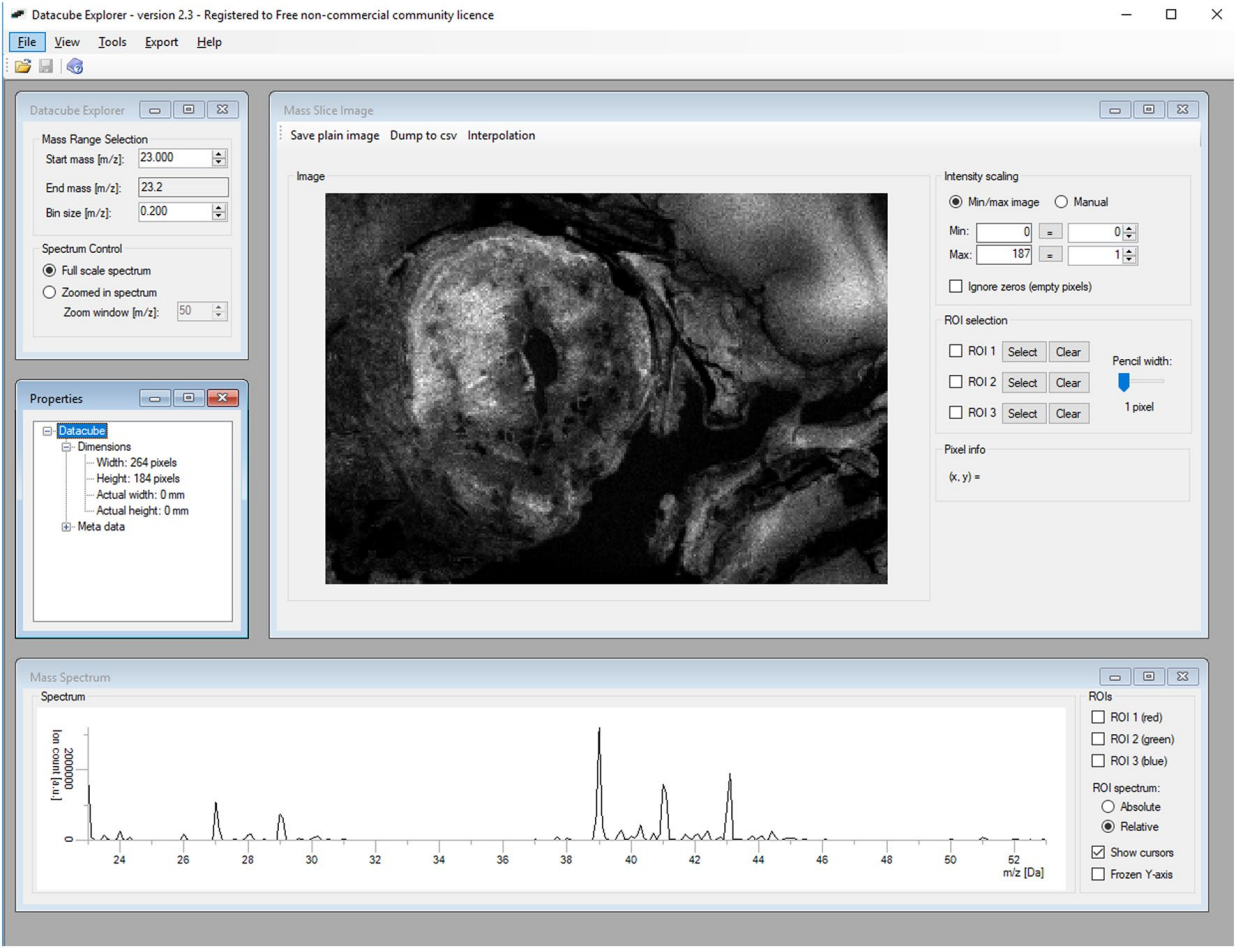
Data set analysis with the DataCube Explorer. The Datacube explorer screen has a clear layout consisting of four individual windows, i.e. “Datacube explorer”, “Mass slice image”, “Properties” and “Mass spectrum”. In the depicted screenshot, a fish eye was analyzed. More details of the DataCube Explorer are given elsewhere [62, 63]

Beside the discussed ones, there are many other valuable programs for analyzing MS imaging data. In 2013, the open MATLAB-based tool OmniSpect was introduced by scientists of the Emory University and the Georgia Institute of Technology, Atlanta, USA [60]. Based on information given in the original report, this software provides key analysis capabilities, accepts input from common MSI data forms, can visualize and compare composite figures, and allows data analysis to be performed remotely using computational resources typically unavailable locally [60]. Sophisticated software tools to process MS images were also developed by international consortia aiming to develop and strengthen technologies and methods for MSI. One such initiative, the COMPUTIS consortia consisting of academic and industrial institutes have launched the DataCube Explorer, SpectViewer, and the EasyReg2D tools [61]. These programs and tools are user-friendly and permit to manage huge data sets quickly and to provide efficient assistance for the visualization and interpretation of data. The DataCube Explorer, for example, is software capable of visualizing datacube format, imzML and the Biomap file format [62]. During data set analysis, information is concisely displayed in four different windows, i.e. ‘The Datacube Explorer Window’, ‘The Mass Slice Image Window’, ‘The Mass Spectrum Window’, and ‘The Properties Windows’ (Fig. 7). In order to use the Datacube Explorer software, a free licence key is required to activate the software. The respective key, a manual for using the program, installation files, and some Datacube data sets can be found at [63]. Although the installation is a bit complicated, this program is an important tool for editing MS data.

**Fig. 8:**
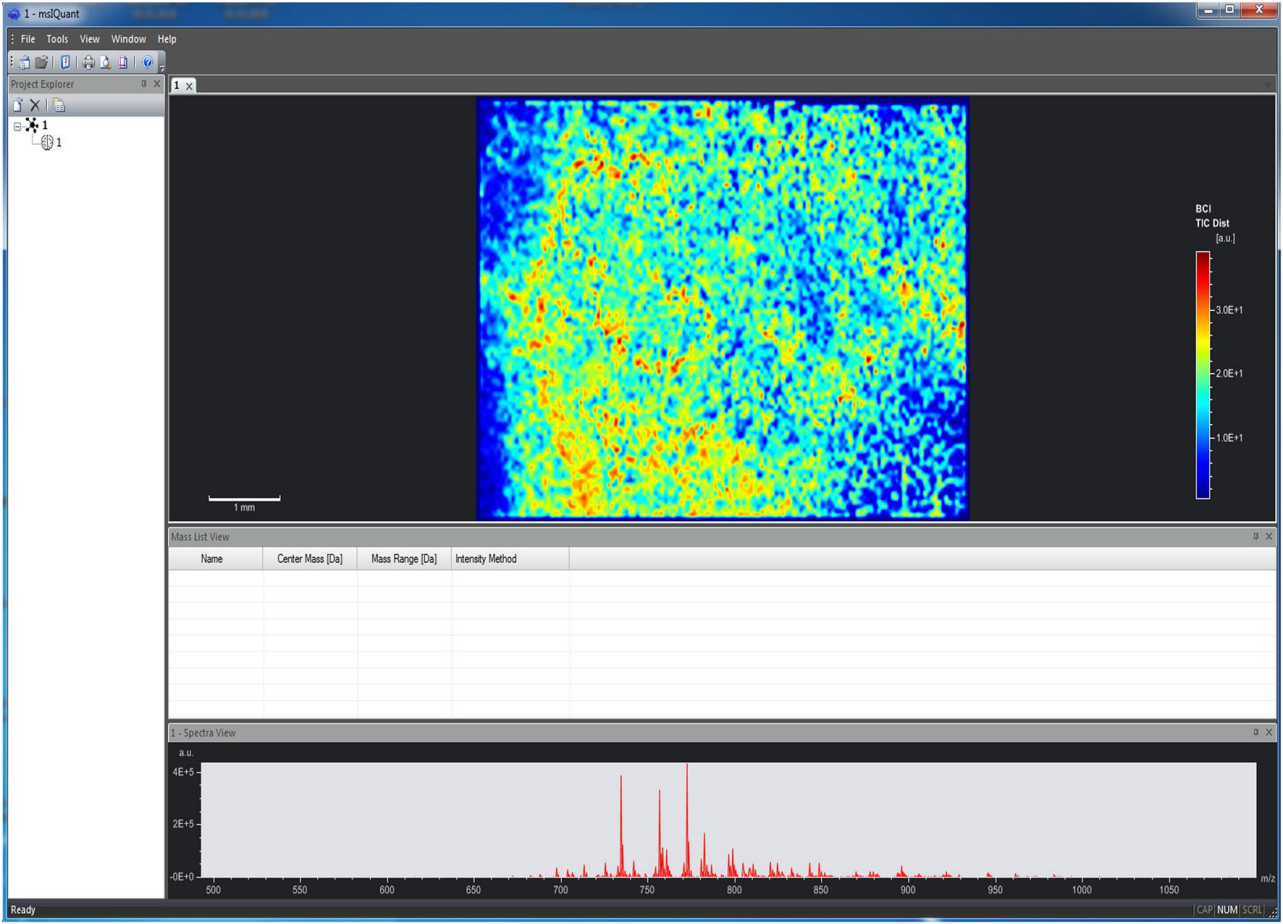
The mass spectrometry imaging software msIQuant. The program msIQuant is an open access, instrument- and manufacturer-independent software for visualization. The graphical user interface of msIQuant has four windows. The “Project Explorer” displays basic information about the experiment, the “Spectra view” displays the average and maximum intensity of spectra measured in the sample, the “Mass list view” provides information about center and mass range, while the “Image view” contains scaled information about the distribution of the selected ion within the sample. The depicted example was taken from the included sample file “Aspergillus”. Details about the software development, necessary data formats, quantitation, and performance of the MSI software are given elsewhere [66]

In addition, other programs or scripts such as Create Target/AnalyzeThis! were launched to assist MSI on vendor-specific devices [64]. Comparable, the ImageJ plugin OpenMIMS is capable to process and analyse images captured with special ion mass spectrometers [65]. Contrarily, the msIQuant quantification software was developed as an image visualization tool enabling fast access, visualization, and analysis of large data sets in an instrument- and manufacturer-independent fashion (Fig. 8) [66]. Although the speed in data processing is impressive, this program has only a limited number of possibilities for evaluating LA-ICP-MS data. Moreover, the main windows of msIQuant are less appealing than those of other programs. Other algorithms, or self-written analysis tools such as Cardinal [67], special MSI.R scripts [68] and massPix [69] are helpful for statistical analysis and interpretation of MS data. In addition, efforts are being made to visualize biological indices calculated from MSI data or to identify special elements or molecules from ion spectra. Examples of such programs are MSIdV [70] and CycloBranch [71, 72]. However, these programs often need previous adaptation before they can be used for a specific application.

## Examples of commercially available and instrument-specific software used in LA-ICP-MS imaging

There are several proprietary or ‘closed source’ software programs available for interactive graphing and data analysis that are frequently used in LA-ICP-MSI laboratories. Commercial programs are protected by copyright and license agreements containing specific ‘terms of use’ that are usually not negotiable. In addition, there are some company-specific software packages developed by manufacturers of LA-ICP-MSI systems, which are intimately linked with respective devices and data formats. Although most of these programs are not specially designed for LA-ICP-MSI data processing, the general usability and the variety of application possibilities have attracted many LA-ICP-MSI end-users. Following, some of the most popular commercially distributed programs and instrument-specific software will be briefly discussed in alphabetical order.

## 4000ImageView

Applied Biosystems (since 2014 acquired by ThermoFisher Scientific) have developed a special image acquisition software for their own 4000 Series Proteomics Analyzers. It is easy to handle and allows the acquisition of MSI files through a seamless integration of existing software. Different versions of this program and additional information are freely available [73].

## HDF-based Image Processing software

The HDF-based Image Processing (HDIP) software was recently launched by Teledyne CETAC Technologies (Omaha, NE, USA). Although there is still little experience with this program, it is advertised by the company as the most powerful LA-ICP-MS imaging software on the market today [74]. It accepts all major ICP-MS file formats as well as raw TOF pilot data and can process imaging data that is stored in the hierarchical data format 5 (HDF5). It is equipped with an extensive toolkit for MS data processing, with a specific emphasis on LA-ICP-MS imaging data. HDIP uses basic and advanced algorithms, which can rapidly process complex LA-ICP-MS data sets. The results can be visualized in 2D and 3D images and exported into many data formats. In addition, it has a number of sophisticated features. The AutoPilot feature processes data fully-autonomously from raw data towards the end-stages of data processing. In the False Color Map Editor, images can be adjusted in regard to transparency, scaling and threshold. In addition, integrated data restoration applications allow the retrieval of better spatial resolution. Moreover, an external calibration tool including a database of standard and certified reference materials is included in the respective software package. Other integrated features of HDIP that should help to analyze or make quick calculations from single or repetitive measurements are the “Replicate Inspection Tool”, “Sum Normalization”, “Channel Calculator”, “Automatic Peak Analysis”, and the “Spreadsheet Tool”. However, the software has not been on the market for long (release date July 3, 2018) and the future will show whether the program fulfills the promises made.

## High Definition Imaging Software

The High Definition Imaging (HDI) software solution (Waters Corporation, Milford, MA) is specifically designed to simplify and streamline the MS imaging workflow. It allows the user to fully integrate all steps into a SYNAPT mass spectrometer imaging trial via a single intuitive interface [75]. The company advertises their software as a powerful yet intuitive software package including all the data analysis and sophisticated statistical tools necessary for fast and efficient data analysis of highly complex imaging data. As such, the application/script is usable and embeddable in many molecular imaging applications that are based on desorption electrospray ionization, matrix-assisted laser desorption/ionization, ion mobility MS, time of flight MS, MS/MS applications, and tandem MS data acquisition using alternating low-energy collision-induced dissociation (MS^E^). The operating system is Windows and the usage requires only medium computer skills. For image generation and manipulation, generated data can be imported into many other software packages including BioMap.

## ImageQuest software

ImageQuest Software (ThermoFisher Scientific, Waltham, MA) was developed to detect, identify and determine the distributions of compounds in a single analysis. The software enables the visualization of imaging data and allows the generation of 2D and 3D element maps. It is specifically designed for Thermo Scientific instruments such as MALDI on LTQ XL and MALDI on LTQ-Orbitrap. A user guide in English language can be freely downloaded from the homepage of ThermoFisher Scientific at [76].

## IMAGEREVEAL

The IMAGEREVEAL MSI data analysis software (Shimadzu, Kyoto, Japan) currently supports the file formats Analyze 7.5, imzML, kbd and the proprietary format IMDX. The actual software of IMAGEREVEAL includes six types of functionality for data analysis. Their availability and function depends on the license selected by the customer. After filling out a form, demonstration software of this program is available [77].

## MALDIVision

MALDIVision from Premier Biosoft (Palo Alto, CA) was developed to visualize and analyze the spatial distribution of individual ions in a tissue section. The possibility to overlay a MALDI image over an optical image makes this program ideal for studies in which respective information should be analyzed in parallel. It allows handling of large imaging data sets, has a comprehensive image display, can import tissue optical image, extract ion images, and can export ion intensity maps in high quality. A free trial can be requested [78].

## MassImager

This software was introduced as a user-friendly, generic, flexible, powerful, and full-featured MSI standalone software tool [79]. It includes three subsystems (i.e. Solution, Visualization, and Intelligence) and is compatible with Microsoft Windows XP/Vista/7/8/10. Two editions for Win32 and Win64 platforms are available. The software is programmed in C++ and suitable for quick reconstruction and analysis of large data sets (“Big data”) and images (dozens of gigabytes). It offers several self-defined operations for visual processing and highly precise regions-of-interest analysis. MassImager has an overall easy work flow and contains some innovative elements including automatic pattern recognition, interactive visualization between ion images and mass spectra, and possibilities for regional spectra calculation. Although it is only commercially available, free trial demo software, including a bilingual user manual in Chinese and English is freely available after registration [80].

## MSImageView

MSImageView from Novartis (Basel, Switzerland) was originally developed for conversion of data acquired with the FlashQuant workstation of ThermoFisher Scientific that was available as an upgrade to the 4000 QTRAP^®^ LC/MS/MS system from SCIEX (Framingham, MA, USA) that are no longer in production. However, MSImageView is still frequently used. It allows exporting images in JPEG and imzML format. A download link for the freeware MSImageView can be found at [81].

## MMSIT

MMSIT, again copyrighted by Novartis (Basel, Switzerland) was developed for automated imaging of samples analyzed on instruments of Applied Biosystems (Voyager STR, Voyager sSRT). In this software MS images are calculated using an optimized baseline correction routine, while drag-and-drop operation allows easy handling of data. A detailed description of program features and a download link are available [82].

## Origin and OriginPro

Origin distributed by OriginLab (OriginLab Corporation, Northampton, MA), is a powerful and fully-featured general data analysis and visualization program used by many customers. It offers features for linear and non-linear curve fitting, model validation, data set comparison, peak and surface fitting, and multi-dimensional data analysis. Moreover, the program includes a number of statistical tests for hypothesis testing, model development and verification. The versatile program can easily be connected with other applications (MATLAB, LabVIEW, Mathematica, Microsoft Excel, and others) [83]. Moreover, Origin supports different output formats (simple columns, 2D and 3D representations), flexible data input, easy data processing, contains a fitting function, and enables the creation of publication quality figures. It should be emphasized that Origin can create templates for repetitive tasks or to perform batch operations, and is easily connectable with other application such as MATLAB, LabVIEW, or Microsoft Excel. Details about the program were recently published elsewhere [84]. OriginPro is an extension of Origin offering advanced analysis tools for peak fitting, surface fitting, statistics, and signal processing [85].

## PMOD

The Peripheral MODule interface (PMOD) from PMOD Technologies LLC (Zürich, Switzerland) is a software package that includes a large variety of modules for quantitative data processing [86]. This software is easy to use and can be integrated into many other operating systems and networks. It offers features for quantitative approaches, handling of calibration factors, and for processing of value units and pertinent geometrical information. Moreover, it includes an extensive statistic interface and a highly flexible set of tools for volumes of interest definition. The program includes a great number of image processing procedures and the PMOD batch pipeline supports fully automatic image processing that can be exported in a large number of formats. Most importantly, PMOD comprises modelling tools in which 50 kinetic model configurations are available. Based on its comprehensive possibilities, PMOD is used for many applications including LA-ICP-MS imaging. To get a first-hand experience with this program, the company offers slimmed down local trail versions with a temporary license. This trail version works for 2 months.

## Quantinetix

Quantinetix from ImaBiotech SAS (Billerica, MA) was developed to quantify and normalize images following MS imaging experiments. It is compatible with data coming from MS systems of different manufacturers including Bruker, SCIEX, ThermoFisher Scientific, and Waters. ImaBiotech and Bruker pursue a common goal in marketing and have signed a software distribution agreement to promote and distribute Quantinetix software. This software is adjustable to common data formats for MS imaging, including imzML, a data format specially developed for the flexible exchange and processing of MSI data [87]. Although there is presently no specific peer review article available dedicated to the software only, several scientific projects have been successfully published with the use of this software. A typical example in which Quantinetix was used for quantitation of a neuropeptide can be found elsewhere [88]. An innovative further development of this program by ImaBiotech is Multimaging. This is a platform which uses multimodal imaging techniques and integrates innovations to overlay imaging results established by immunohistochemistry, immune-staining, MSI, and other imaging methods.

## SCiLS Lab

SCiLS Lab (SCiLS, Bremen, Germany) is an MSI imaging software for data handling and manipulation. It has numerous visualization options and can process sets of virtually unlimited size. SCiLS Lab offers the opportunity for classification model calculation, co-localization analysis, comparative analysis for uncovering discriminative *m/z* markers, and to import lists of masses from public or private databases [89]. In addition, the program allows batch processing and export of images and tables to Microsoft Office applications. Free trial versions of this software are available [90].

## TissueView

The TissueView software was developed by ThermoFisher Scientific as a simple solution for processing and displaying data from MSI experiments, particularly from ThermoFisher devices (e.g. 4700 and 4800 MALDI TOF/TOF analyzers, QStar Pulsar i System, QSTAR^®^XL hybrid LC/MS/MS system, QSTAR^®^ Elite platform). Although the software is optimized for proteins and drugs, it should in principle allow analysis of other molecules within biological tissue or to export color scales, signal intensities, and other semi-quantitative information on ions and small molecules.

## Pros and cons of commercially available and instrument-specific software

Often special data analysis programs are bundled with mass imaging devices, which makes data handling easy. These programs are generally feature-rich and allow the handling of a large variety of different measurements. In addition, the vendor often provides support for application problems. On the downside, the complete design and quality assurance testing is handled by the vendor only, preventing for example the fast discovery and fixing of software bugs. Moreover, licensing fees as well as updates can become costly. Since these multi-functional programs are developed for a wide audience using manifold applications and not specially tailored for LA-ICP-MS users, they often include a high number of superfluous features that unnecessarily consume computing resources. On the other side, modification or extension in these elaborated software solutions are generally not possible, preventing adjustments in the workflow and data management. Sometimes these commercial programs have substantial limitations in converting data into other formats, which inhibits embedding and processing of data into other software routines and data exchange. Last but not least, such programs may include security or technical solutions not conforming to the internal guidelines of the institution in which the software is used, such as in clinics. Therefore, some users prefer free and open-source software or even develop their own, highly specialized, programs that are optimized for the workflow and requirements of the user. In most cases, these solutions are safer, stable, and more resistant to malware or attacks compared to commercial software. In addition, they are more reliable in protecting privacy giving users more control over their own hardware and data.

## Conclusions and perspectives

Suitable software for visualization of LA-ICP-MS imaging data are a prerequisite for simple representation of the vast amounts of raw data generated by this technique. Currently, there is a large selection of such programs.

Commercial software usually requires the purchase of a license, which can range from a few hundred dollars to several hundred or thousand dollars. A few of them are user-friendly, generic, flexible, powerful and suitable to create high-quality and meaningful images. In addition, preformatted software supplied with equipment allows quick operation. On the other hand, these software tools were developed for specific applications or selected mass spectrometers. This makes it difficult to adapt these programs for more specific applications.

If a laboratory only needs a few standardized evaluation routines for daily data processing, simple in-house software, which is integrated into a general workflow, is already suitable to fulfill all needs of the user.

Open source software allows simple customization to the needs of the user. Furthermore, developers can permanently contribute modifications into the source code to improve the program continually. However, this is only possible if sufficient knowledge in writing such application routines is present. Unfortunately, learning a programming language like Java, Python or R is not necessarily easy and most operators therefore prefer to use commercial software.

As discussed, several programs for evaluation and visualization of LA-ICP-MS data were developed in the scientific community. Surely, these programs are not always suitable for handling large data sets. In addition, algorithms for quality control and quality assurance are generally missing in such programs. In the most severe case this can lead to erroneous analysis of measured data sets. Moreover, it is questionable whether the different self-made programs will generate comparable results from an identical data set. Sloppily programmed software also may generate post-analytical errors and thus faulty interpretations. Therefore, the disclosure of software code under open source licenses, and thorough documentation of their installation and use greatly supports the development of LA-ICP-MSI programs.

To sum up, there is a huge diversity of complementary software for processing and visualizing LA-ICP-MSI data. The different file formats impede the exchange of data and their evaluation on different platforms. It would therefore be desirable to define a uniform data format, in which all collected LA-ICP-MS data are presented. The use of such a community data format would make it possible to exchange MSI raw data easily, generate robust graphical and statistical tools for data evaluation and thus improve the overall quality of respective visualization programs.

## Abbreviations

ELAI: Excel Laser Ablation Imaging
HDI: High Definition Imaging
IMAGENA: IMAgeGENeration and Analysis
ISIDAS: Interactive Spectral Imaging Data Analysis Software
LA-ICP-MS: laser ablation inductively coupled plasma mass spectrometry
MS: mass spectrometry
MSI: mass spectrometry imaging
PMOD: Peripheral MODule interface
SILLS: Signal Integration for Laboratory Laser Systems
SMAK: Sam’s Microprobe Analysis Kit
